# Higher resources decrease fluctuating selection during host–parasite coevolution

**DOI:** 10.1111/ele.12337

**Published:** 2014-08-28

**Authors:** Laura Lopez Pascua, Alex R Hall, Alex Best, Andrew D Morgan, Mike Boots, Angus Buckling

**Affiliations:** 1 Oxford Regional Molecular Genetics Laboratory, Oxford University Hospitals NHS TrustOxford, UK; 2 Institute of Integrative Biology, ETH Zürich8092, Zürich, Switzerland; 3 School of Mathematics and Statistics, University of SheffieldSheffield, S3 7RH, UK; 4 Institute of Evolutionary Biology, The University of EdinburghEdinburgh, EH9 3JT, UK; 5 Biosciences, University of ExeterPenryn, Cornwall, TR10 9EZ, UK

**Keywords:** Adaptive dynamics, bacteria, experimental evolution, virus

## Abstract

We still know very little about how the environment influences coevolutionary dynamics. Here, we investigated both theoretically and empirically how nutrient availability affects the relative extent of escalation of resistance and infectivity (arms race dynamic; ARD) and fluctuating selection (fluctuating selection dynamic; FSD) in experimentally coevolving populations of bacteria and viruses. By comparing interactions between clones of bacteria and viruses both within- and between-time points, we show that increasing nutrient availability resulted in coevolution shifting from FSD, with fluctuations in average infectivity and resistance ranges over time, to ARD. Our model shows that range fluctuations with lower nutrient availability can be explained both by elevated costs of resistance (a direct effect of nutrient availability), and reduced benefits of resistance when population sizes of hosts and parasites are lower (an indirect effect). Nutrient availability can therefore predictably and generally affect qualitative coevolutionary dynamics by both direct and indirect (mediated through ecological feedbacks) effects on costs of resistance.

## Introduction

Antagonistic coevolution between hosts and parasites, the reciprocal evolution of host defence and parasite counter-defence, has far-reaching consequences for the evolution of genetic and ecological diversity (Thompson 2005), parasite virulence (Anderson & May 1982), sexual reproduction (Hamilton *et al.* 1990), mutation rates (Pal *et al.* 2007) and population dynamics (Buckling & Hodgson 2007). Crucial to the impact of coevolution is the extent to which selection on infectivity and resistance traits fluctuates through time, since a fluctuating selection dynamic (FSD) plays a key role in the maintenance of diversity and the persistence of coevolution (Hamilton *et al.* 1990; Sasaki 2000; Agrawal & Lively 2002). It is well known that the extent of FSD is in part determined by the genetic specificity between host and parasite. At one extreme (high and equal specificity), each parasite genotype can only infect a single host genotype, hence genotypes are highly specialised (matching alleles model; MAM) (Frank 1993), and their frequencies will fluctuate through time. At the other extreme (variable specificity), parasites can evolve to be generalists, infecting all host genotypes, as well as specialists, and, likewise, hosts can evolve to be both generalists and specialists. This is traditionally modelled as a Gene for Gene model (GFGM) (Flor 1956; Sasaki 2000; Thrall & Burdon 2003), but can also be captured by a continuous ‘Range’ phenotypic model (Best *et al.* 2010). In the absence of costs associated with generalism, there will be selection for increasing resistance and infectivity ranges, resulting in arms race dynamics (ARD). However, if there are costs associated with generalism, range evolution will be constrained and FSD becomes possible.

The FSD in gene-for-gene type models can be of two types. First, fluctuations in the frequencies of genotypes with similar ranges, but able to infect/resist different enemy genotypes (specialism FSD); analogous to FSD resulting from MAM specificity (Agrawal & Lively 2002). Second, fluctuations in the magnitude of infectivity and resistance ranges (Sasaki 2000; Best *et al.* 2010) (range FSD). Costs may also lead to the stable persistence of a number of hosts and parasites with different ranges (Best *et al.* 2010): static rather than fluctuating diversity. There is a general perception that environmental conditions will influence the outcome of coevolution between hosts and parasites but we have little understanding of how different environmental factors impact on the likelihood of FSD versus ARD. Here, we investigate the impact of nutrient availability, which can affect costs and benefits of resistance (Hochberg & van Baalen 1998; Lopez-Pascua & Buckling 2008) on the extent of ARD versus FSD in coevolving populations of bacteria and viruses.

Recent work using coevolving populations of bacteria and viruses (bacteriophage; phage) is supportive of the role of costs in determining the relative importance of ARD versus FSD. For example, the early stages of coevolution between the bacterium *Pseudomonas fluorescens* SBW25 and phage SBW25ϕ2 in nutrient-rich media are characterised by ARD, with FSD becoming increasingly important through time (Hall *et al.* 2011). This increase in FSD correlates with increased costs associated with increased bacterial resistance ranges (Hall *et al.* 2011). Moreover, coevolution between the same bacteria and phage in soil microcosms is characterised by FSD rather than ARD even at early stages, and costs associated with increased resistance range are greatly elevated in soil compared to nutrient-rich laboratory media (Gomez & Buckling 2011).

Costs of resistance have the potential to be affected by a wide range of environmental conditions, including nutrient availability. Theoretical and empirical studies suggest complex effects of nutrient availability on host–parasite antagonistic coevolution, but a general finding is that increased nutrients tend to result in the evolution of elevated resistance (Hochberg & van Baalen 1998; Bohannan & Lenski 2000a,b; Forde *et al.* 2004, 2007, 2008; Lopez-Pascua & Buckling 2008; Lopez-Pascua *et al.* 2010; Boots 2011; Harrison *et al.* 2013). There are two general reasons for this, which have proved hard to tease apart (Lopez-Pascua & Buckling 2008). First, costs of resistance can be reduced when there is less competition for resources. Second, increased nutrient availability increases host population sizes, and hence host–parasite encounter rates, increasing selection for resistance. As a consequence of increased host resistance with increased nutrient availability, selection for elevated parasite infectivity is likely to increase in turn (Hochberg & van Baalen 1998; Lopez-Pascua & Buckling 2008). An intriguing possibility is that increasing nutrient availability has the potential to shift a dynamic from FSD to ARD.

Here, we provide the first empirical and theoretical tests of this hypothesis. We measured the extent of ARD and the different FSDs between *P. fluorescens* SBW25 and ϕ2 coevolved under high and low nutrient conditions by measuring interactions between multiple bacteria and phage clones within and between time points, within communities. We carried out these assays for communities in which coevolution had previously been determined by measuring resistance and infectivity at the population level only (Lopez-Pascua & Buckling 2008). This distinction between population level assays and clonal level assays (this study) is crucial, because only clonal level assays can ensure discrimination between different coevolutionary dynamics. We then build a mathematical model of the coevolution of host and parasite range that explicitly includes the ecological dynamics of the system. With this we examine the impacts of both the direct effects of changes in costs and the indirect effects of nutrient supply on population size on FSD versus ARD.

## Materials and Methods

### Selection experiment

We used bacteria and phage that had been coevolved for a previous study (Lopez-Pascua & Buckling 2008). Selection lines were initiated by inoculating 6 mL high (or low) nutrient M9KB medium (M9 salt solution supplemented with 10 (1) gL^−1^ glycerol and 20 (2) gL^−1^ proteose peptone) with ∼ 10^8^ cells of *P. fluorescens* SBW25 and ∼ 10^5^ particles of SBW25Φ2. Six replicate selection lines per nutrient treatment were maintained by serial transfer, with a 100-fold dilution every 48 h. Tubes were incubated at 28 °C static, and vortexed for 1 min to homogenise the culture prior to each transfer to fresh media. Each selection line was maintained for 12 transfers, and a sample of each was frozen at −80 °C in 20% v : v glycerol every two transfers.

### Isolating coevolved bacteria and phage

To measure changes in infectivity and resistance over time, we isolated 20 independent phage plaques and bacterial colonies (clonal isolates) from the frozen populations at transfers 2, 4, 6, 8, 10 and 12 for each of the 12 replicates. Bacteria were isolated by plating samples onto M9KB agar plates following overnight growth in liquid M9KB media at 28 °C, which were then incubated overnight at 28 °C. Colonies were then inoculated into liquid M9KB overnight for subsequent resistance assays. A sample of each culture was also frozen at −80 °C in 25% v : v glycerol. Phages were extracted from the same frozen samples by adding 10% chloroform to the reconditioned culture, vortexing and centrifuging at 13 000 rpm (13800 g) for 2 min, serially diluting and spotting onto M9KB agar (6%) containing exponentially growing ancestral bacteria, prior to overnight incubation and picking independent plaques. Each plaque was then amplified on ancestral bacteria before storage at 4 °C. While this method of phage isolation will bias against phage clones that are unable to grow on ancestral bacteria, a previous study showed such phage phenotypes to be at low frequencies (Hall *et al.* 2011). This procedure yielded a total of 120 host and 120 phage clonal isolates for each of the 12 coevolving communities.

### Infectivity and resistance assays

We tested for infectivity/resistance in every phage/bacteria combination within each of the twelve replicate populations (120 phages × 120 bacteria = 14 400 assays per population). For each assay, 1 μL of phage stock was added to a lawn of the relevant host bacteria growing on soft M9KB agar. Phages were scored as infective if plaques were visible after incubation at 28 °C for 24 h. Infectivity at each combination of phage time (the transfer that phages were isolated from) and bacteria time (the transfer that bacteria were isolated from) was calculated as the proportion of successful infections (*n *=* *400).

### Statistical methods

Coevolutionary dynamics are unlikely to follow one prescribed model: ARD, range FSD and specialism FSD may operate to different extents within one coevolving community. Partitioning total evolutionary change into the three coevolutionary dynamics is problematic because the selection is not attributable to the interacting enemy; drift and experimental error are all likely to contribute to observed changes, and some observed changes will be consistent with more than one coevolutionary dynamic. However, unique signatures of the different dynamics can be measured and compared between communities that coevolved in different environmental conditions (Fig. S1).

*Arms race dynamics* are characterised by increasing resistance and infectivity ranges through time, such that parasite infectivity to contemporary hosts should increase from past to future parasite populations, and host resistance to contemporary parasites should increase from past to future host populations. This can be inferred across the entire time series for a given population from the association between phage infectivity and time shift, where time shift is the difference between the transfer that bacteria and phages were sampled from (Rode *et al.* 2011; Blanquart & Gandon 2013). For example, when phage particles from transfer 4 are tested against bacteria from transfer 10, time shift = 10–4 = 6. Under ARD, we expect a negative slope of average infectivity against time shift. We tested this association in high and low nutrient treatments by taking the average infectivity at each combination of phage time, bacteria time and population, before fitting a linear model with infectivity against phage time and time shift, plus population (random) and its interactions with both fixed effects, thereby fitting six independent estimates for the slope of infectivity against time shift, one for each population.

*Range FSD* implies that infectivity/resistance ranges both increase and decrease through time. This results in a main effect of phage time or bacteria time fitted as categorical predictors of average infectivity in interactions between phages and bacteria across the time series. In our data set, the main effects varied among populations in both nutrient treatments (population × phage time or bacteria time effects). We, therefore, tested for range FSD within each population by fitting a model of phage infectivity against phage time, bacteria time and their interaction, taking the mean square for each main effect as an estimate of the strength of range FSD for infectivity and resistance, before testing for an average effect of nutrient supply by a *t*-test. Because significant main effects of phage time and bacteria time can also result from ARD, range FSD is only clearly demonstrated in a given population by a main effect of phage time or bacteria time in the absence of directional changes as inferred from the time shift effect described above.

*Specialism FSD* results in phages and hosts from different time points being specialised to infect/resist different sets of hosts/phages, and a significant phage time × bacteria time interaction in determining population-level infectivity. Because this can also stem from random processes such as sampling error or genetic drift, specialism FSD can only be unambiguosuly shown if parasites (or hosts) are best adapted to their contemporary enemies relative to past and future enemies. In the absence of this pattern (as was the case for this data set), specialism FSD, which is ultimately driven by negative frequency-dependent selection, could still be manifest at the level of individual clones if, in a given population at a single time point, there is variation among clones in their ability to infect/resist hosts/phages from the past and the future. By contrast, under ARD or range FSD, the relative susceptibility/infectivity of hosts/phages from different time points would not vary qualitatively among clones. To test this, for five time points (4, 6, 8, 10), we took the average infectivity of each of the 20 phage clones against hosts from the past, present and future. We then calculated the inconsistency component of the phage clone × time (past/present/future bacteria) interaction, calculated as Σ(σ_e1_σ_e2_(1 − 1ρ_e1e2_)), where σ_e1_ and σ_e2_ are the standard deviations of infectivity across time points for two clones, and ρ_e1e2_ is the correlation between the two clones across time points (Bell 1990; Venail *et al.* 2008). A similar calculation was carried out for resistance inconsistency, where the resistance of each clone from a time point was calculated against past, present and future phages.

### Modelling

We build on the modelling framework presented in Best *et al*. (2010) which developed an ecologically explicit coevolutionary model where host resistance and parasite infectivity range coevolve as continuous traits. The model includes both epidemiological and ecological dynamics and is therefore an appropriate framework to examine the role that variation in costs associated with increased resistance range for hosts or infectivity for parasites plays in the prevalence of FSD relative to ARD. Since the model has intrinsic ecological dynamics with an emergent carrying capacity and disease equilibrium dynamics, we can also independently examine the influence of reduced intra-specific competition and higher population sizes associated with increased nutrient concentrations. The focus of Best *et al*. (2010) was how and when ‘static’ genetic variation is generated by epidemiological feedbacks while here our focus is on how changes in costs and population size influence the chance of cycles in range (range FSD) or the fixation of maximal range (ARD). Note that this modelling framework does not allow specialism FSD, but we consider this an acceptable limitation, given that specialism FSD played a very minor role in the empirical coevolutionary dynamics.

Following Best *et al*. (2010), we use a standard Susceptible-Infected (SI) framework (Anderson & May 1981), with the ecological dynamics of susceptible hosts (*X*) and infected hosts (*Y*) governed by the following equations,(1)
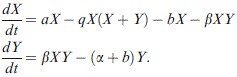


**Figure 1:**
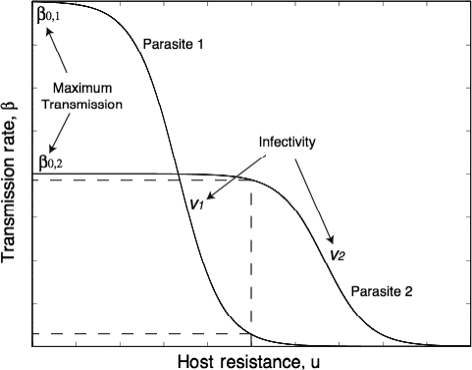
Illustration of the infection function used in the theoretical model in equation (2). Parasite 1 has a small infection range (v1) but achieves high transmission rates against those hosts it can infect. In contrast, parasite 2 has a large infection range (v2) but infects these hosts at a lower rate. Hosts with low resistance ranges (u) will be infected by more parasite types but are assumed to have higher birth rates.

Uninfected hosts reproduce at rate *a*, which is reduced due to crowding by a factor *q*, and have natural death rate *b*. The transmission coefficient of the infection is β, and infected hosts have an additional mortality rate due to infection, α. We assume an infection function such that there is a continuous smooth range of hosts that can be infected by a particular parasite strain and that the narrower the range, the higher the transmission rate of the parasite strain. There is, therefore, the phenotypic assumption of a continuous gene-for-gene model with costs (high range leads to lower infectivity in any particular host). The function is given by(2)
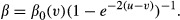
where *u* and *v* denote the host resistance range and parasite infection range respectively, and is best understood from Fig. 1. Here β_0_(*v*) is the maximum transmission rate that a parasite with infectivity range *v* can achieve. A host with a higher *u* is able to prevent infection from stronger parasites (corresponding to resistance to a broader range of potential parasite types), and similarly a parasite with a higher *v* is able to infect stronger hosts (similarly, corresponding to an ability to infect a broader range of host types). The final assumptions are that hosts that have high resistance to a large range of parasites pay a cost in terms of their birth rate, *a*, and parasites with a broad infectivity range have reduced maximum transmission, *β*_0_(*v*).

We analyse the model within an evolutionary invasion (‘adaptive dynamics’) framework (Dieckmann & Law 1996; Geritz *et al.* 1998) where we assume that a rare mutant host (*u*_*m*_) or parasite strain (*v*_*m*_) with a small phenotypic difference attempts to invade a system with resident host and parasite strains (*u* and *v*) which are at an equilibrium of their epidemiological dynamics in (1). The success of these mutants are given by their invasion fitnesses that are found to be as follows:(3)
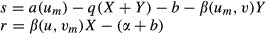
for the host and parasite respectively, where X and Y are the resident equilibria found from solving (1). If *s *>* *0 (*r *>* *0), then the mutant host (parasite) will successfully invade and potentially replace the resident. Through a series of mutations and substitutions, the resistance and infectivity ranges will coevolve until a (potentially temporary) stopping point, or ‘evolutionarily singular point’ is reached when the fitness gradients are simultaneously zero. Here the coevolutionary behaviour depends on a number of second-order terms (Geritz *et al.* 1998). Our focus here is on ‘convergence stability’, i.e. whether the singular point is a coevolutionary attractor but in particular we will look for ‘Hopf bifurcations’ where a singular point loses convergence stability in such a way that coevolutionary cycles are generated: this is the criteria for range FSD.

Convergence stable singular points are found through mathematical analysis of the model (see Best *et al.* 2010 for more details). The behaviour of this point, as a particular parameter is varied, is investigated using the AUTO continuation software (Doedel *et al.* 2008). The software studies the convergence stability of any singular points as small perturbations are made to a chosen parameter, in particular identifying the location and stability of the singular points and any bifurcations. For illustration, we also run numerical simulations of the coevolutionary process for chosen parameter values in the C programming language. N potential host and parasite strains are initiated with the densities of all but one of each population is initially set to zero. The epidemiological dynamics of (1) are run for sufficient time such that the system will be approaching its equilibrium. A host or parasite strain is then selected (weighted by population size and subject to demographic stochasticity (Dieckmann & Law 1996)) to produce a mutant one strain ‘up’ or ‘down’ at low density. The epidemiological dynamics are then run again for sufficient time and a new mutant is selected. Before each mutation, any strains whose density is below some low threshold are assumed to be extinct.

## Results

### Bacteria-phage coevolution

**Figure 2:**
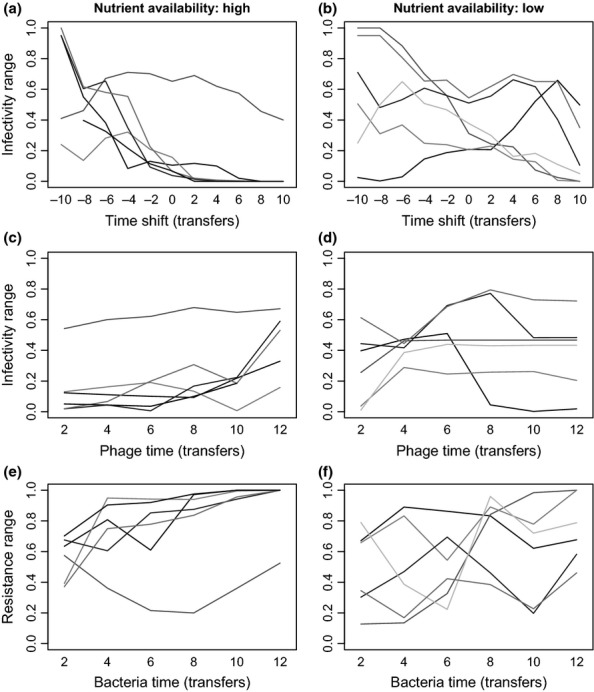
Temporal changes in mean infectivity and resistance ranges through time under high (a, c, e) and low (b, d, f) nutrient availability. Individual lines show the six replicates per treatment. Data are plotted both as a function of time point from which bacteria or phages were isolated, and as a function of the difference in time point between phages and bacteria (time shift).

There was a strong tendency for phages to be more infective against bacteria from the past than the future on average in communities evolved in our high-nutrient treatment (linear time shift effect: *F*_1,5_=22.29, *P *=* *0.002; Fig. 2a), but not in the low-nutrient treatment (*F*_1,5_=3.59, *P *=* *0.11; Fig. 2b). This association, indicating ARD, held for five out of six high-nutrient communities but only two out of six low-nutrient communities (*P* < α = 0.05 after sequential Bonferroni correction). Although the average linear effect of the time shift was stronger at high nutrient supply, infectivity and resistance varied across time points to a similar extent in both treatments on average (categorical effects of phage time and bacteria time in communities from high vs low nutrient supply – phage time: Welch's *t*_10_* *=* *−0.58, *P *=* *0.58; bacteria time: *t*_5.74_* *=* *−0.89, *P *=* *0.41; Fig. 2c–f). Indeed, three out of six low-nutrient communities showed significant variation in both phage infectivity and bacterial resistance across time points but no significant effect of time shift (Table S1). This indicates range FSD: average host range of phages or resistance range of bacteria varied significantly but non-directionally over the time series in these populations. By contrast, there was only one high-nutrient community where bacteria time had a significant but non-directional effect (‘U’-shaped in Fig. 2e).

We next asked whether greater range FSD at low nutrient supply was associated with a stronger tendency for phages or bacteria to be specialised to enemies from particular time points. This would result in a stronger phage time × bacteria time interaction, but the mean square for this term in high- and low-nutrient populations was no different on average (Welch's *t*_6.45_* *=* *0.50, *P *=* *0.64). Although this does not exclude the possibility of specialism FSD, it does not support a difference in the degree of specialisation between high- and low-nutrient supply treatments.

Specialism FSD can still be manifest at the level of individual clonal isolates even if it is not detectable at the population level. For example, a lack of significant variation of population-level performance across past, present and future enemies does not exclude the possibility that different genotypes within the population show qualitatively different reaction norms across enemies from different time points. Therefore, we tested for variation among clonal isolates within time points in their ability to infect/resist hosts from the past, present and future by calculating inconsistency; a measure of within-population specialisation of phage and bacteria clonal isolates to each other. Resistance inconsistency was significantly higher in the low nutrient treatment (*P *<* *0.03), but infectivity inconsistency did not differ between treatments (*P *=* *0.09). Note that levels of inconsistency represented a tiny fraction of the total explained variance in the data set (less than 1%), suggesting that specialism FSD played a small role in observed changes. Nevertheless, the data are consistent with both greater range and specialism FSD for bacteria under low nutrient conditions.

**Figure 3:**
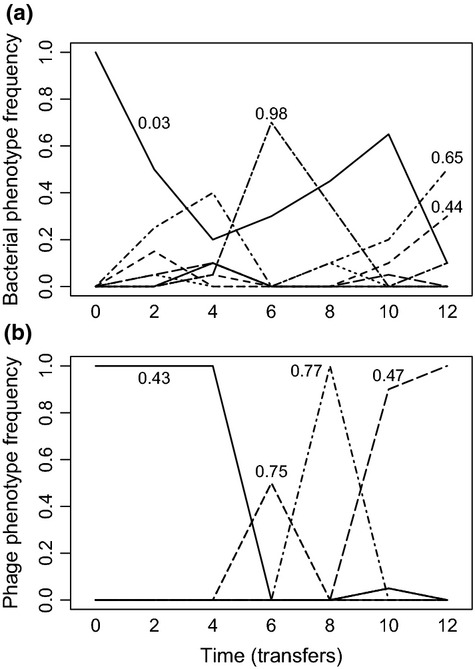
Temporal dynamics of different bacteria (a) and phage (b) phenotypes (based on cluster analyses with 80% similarity) of a single community evolved under low nutrient conditions. Numbers associated with the dominant phenotypes indicate their resistance/infectivity ranges (i.e. proportion of clonal isolates bacteria could resist/phage could infect).

**Figure 4:**
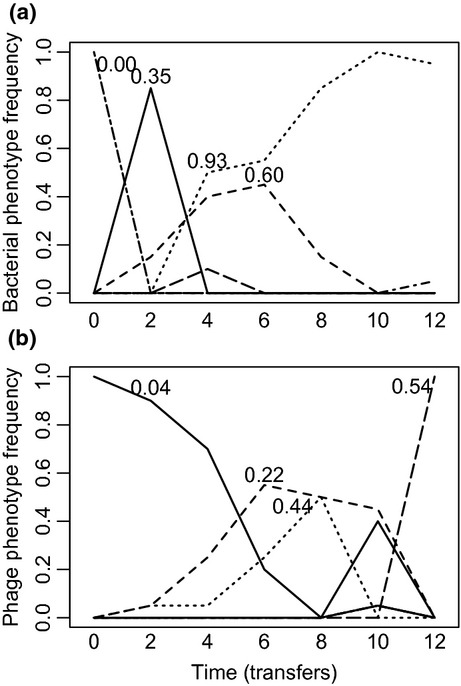
Temporal dynamics of different bacteria (a) and phage (b) phenotypes (based on cluster analyses with 80% similarity) of a single community evolved under high nutrient conditions. Numbers associated with the dominant phenotypes indicate their resistance/infectivity ranges (i.e. proportion of clonal isolates bacteria could resist/phage could infect).

To more clearly visualise these results, we quantified the frequency of individual bacteria and phage phenotypes through time in each population by clustering bacteria and phage clonal isolates into discrete phenotypic complexes (using a squared Euclidean similarity of 80%). The clearest examples from each of the high and low nutrient treatments are shown in Figs3 and 4. In high nutrient media, we typically observed time-lagged directional coevolution towards generalism. The ancestral bacterium, which was sensitive to the ancestral phage, was rapidly replaced by a mutant resistant to the ancestral phage. A phage that could infect this mutant subsequently evolved. This process occurred repeatedly throughout the short experiment. By contrast, although generalists readily evolved in the low nutrient media, they were frequently replaced by phenotypes with narrower resistance and infectivity ranges.

### Modelling

**Figure 5:**
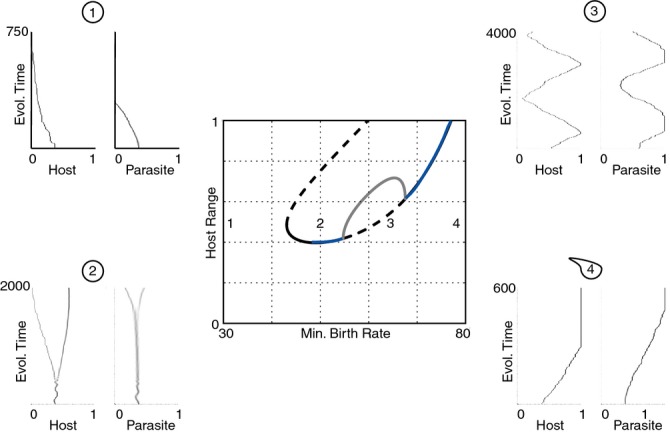
Bifurcation diagram showing the location and stability of host resistance range as the minimum birth rate is varied. Solid black lines denote convergence stable ‘ESS’ points, dashed lines unstable points, blue lines convergence stable but evolutionarily unstable ‘branching’ points and grey lines maximum limit of cycles. Example coevolutionary simulations are also shown for four scenarios (host range on the left, parasite range on the right). In region 1 there is no singular point and both host and parasite minimise their ranges. To the right, stable and unstable intermediate singular points emerge. The stable singular point is initially an ESS but then becomes a branching point in region 2, producing coexisting host (then parasite) strains. A Hopf bifurcation and cycles emerge in region 3. Cycles stop and ranges are maximised in region 4.
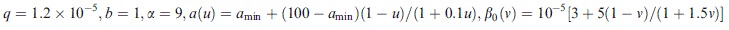

**Figure 6:**
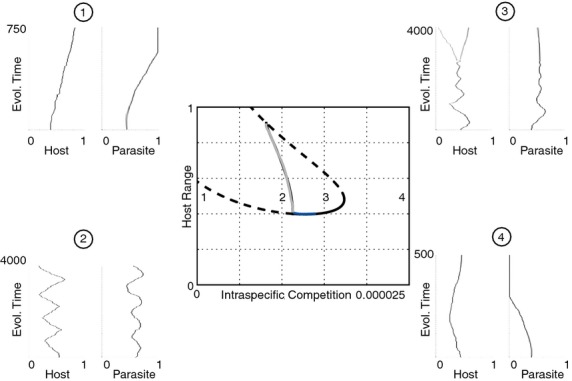
Bifurcation diagram showing the location and stability of host resistance range as competition is varied. Solid black lines denote convergence stable ‘ESS’ points, dashed lines unstable points, blue lines convergence stable but evolutionarily unstable ‘branching’ points and grey lines maximum limit of cycles. Examples of coevolutionary simulations are also shown for four scenarios (host range on the left, parasite range on the right). In region 1 there is no singular point and both host and parasite minimise their range. To the left stable and unstable intermediate singular points emerge. The stable singular point is initially an ESS but becomes a branching point in region 2, producing coexisting host (then parasite) strains. A Hopf bifurcation then occurs and cycles emerge in region 3. These cycles continue until a ‘homoclinic’ orbit where the cycles intersect the unstable singular point. There is no stable singular point in region 4 and ranges are maximised.
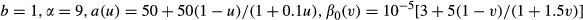

For clarity, we model the effect of resource on the carrying capacity and costs independently. The degree of costs has important implications to the qualitative outcome of the coevolutionary dynamics between the host and the parasite. At high costs, range in both the host and parasite is minimised (region 1 in Fig. 5). As the costs reduce, there is the potential for stable investment at intermediate range or diversification to the stable coexistence of hosts and parasites with multiple ranges (region 2). Then we find cyclic changes in range characteristic of range FSD (region 3), followed by low costs with fixation of maximal host and parasite range (ARD) (region 4). When we model the effect of nutrient supply on population size through a reduction in intra-specific competition leading to a higher carrying capacity, there is fixation of the maximum range (ARD) at the highest population size (region 1 in Fig. 6). This is followed by cycles in range (region 2), diversification in range and intermediate investment as competition increases (region 3). Finally, as population sizes fall beyond this, both host and parasite range are minimised (region 4). Therefore, it is clear from our modelling that high nutrient supply will select for ARD through its effects on reducing costs of resistance and increasing population size.

## Discussion

We measured interactions between clones of bacteria and viruses coevolved under high and low nutrient conditions and developed a general mathematical model. This allowed us to test the novel hypothesis that increasing nutrients shift host–parasite coevolutionary dynamics away from fluctuating selection dynamics to arms race dynamics (FSD and ARD respectively). Both our model and the empirical results support this hypothesis. Our empirical results also suggest greater specialism FSD with lower nutrient availability, although specialism FSD explained a tiny amount of the variation in coevolutionary dynamics, and hence this outcome was not addressed using our modelling framework. The modelling emphasises that range fluctuations with lower nutrient availability can be explained both by elevated costs of resistance, as well as reduced benefits of resistance when population sizes of hosts and parasites are lower. Furthermore, other outcomes than the two dynamics seen in our experiments (i.e. ARD and range FSD) are predicted in the theoretical model when nutrient availability is reduced further, including static range polymorphisms and parasite extinction. Therefore, our key result is that nutrient availability impacts qualitatively on coevolutionary host–parasite dynamics through a direct effect on costs of resistance and an indirect effect through ecological feedback on the benefits of resistance.

Nutrient availability is likely to have complex effects on coevolutionary dynamics, but previous work has identified two effects that are likely to play an important role: lower costs of resistance and increased host–parasite encounter rates with increasing nutrients (Hochberg & van Baalen 1998; Lopez-Pascua & Buckling 2008; Boots 2011). In order to understand their impacts in detail, we therefore modelled these effects independently. Previous theoretical work has investigated the impact of costs of increasing resistance (and infectivity) ranges on coevolutionary dynamics and found, as we have, that increasing costs can shift coevolution from an ARD to a range FSD (Sasaki 2000). This occurs because low costs of resistance resulting from high nutrient availability result in resistance and infectivity reciprocally increasing until maximal investment is reached. However, under lower nutrient availability and hence higher costs of resistance, resistance and infectivity increase until the cost of resistance outweigh the benefits. This results in selection for lower resistance, and in turn, given that there are also costs associated with increasing infectivity, lower infectivity ranges. The cycle is then repeated. The effect of reduced density on the transition from FSD to ARD has not previously been investigated, and we modelled this by assuming higher nutrient availability reduced intraspecific competition and hence equilibrium population densities. The effect of increasing density also shifted dynamics from range FSD to ARD for the same qualitative reasons as above: high density increased encounter rates and hence reduced the costs, relative to the benefits, of resistance. In this experimental system, we have observed that increasing nutrients both reduces costs of resistance and increases density (Lopez-Pascua & Buckling 2008), hence both processes are likely to have contributed to a shift from range FSD to ARD in our experiment. Although not seen in our experimental populations, this same process can result in static diversity where there are multiple phenotypes of hosts and parasites with different resistance and infectivity ranges coexisting (Best *et al.* 2010; Boots *et al.* 2014).

Our empirical results highlight the benefits of assaying resistance/infectivity at the level of individuals in order to understand coevolutionary dynamics. In a previous study, the same populations were assayed for resistance/infectivity at the level of whole populations, but we were only able to detect that nutrients increased the rate of ARD coevolution (Lopez-Pascua & Buckling 2008). While our analyses of clonal data are consistent with this view (the extent of ARD was reduced under low nutrient conditions), we were also able to detect both range and specialism FSD. In the absence of consistently greater performance of hosts or parasites on contemporary, rather than past or future, enemies (Gomez & Buckling 2011), specialism FSD can only be detected with clonal rather than population level data. However, it is important to note that range FSD was obscured with the population-level data because broad infectivity ranges in a population can be achieved by a relatively low frequency of generalist clones. Population level assays can therefore obscure fluctuations in selection for generalism.

One inherent problem with disentangling coevolutionary dynamics using time shift experiments is ruling out the effects of sampling error and genetic drift (Bell 2010). For example, it could be argued that some of the range FSD we observed in communities from our low-nutrient treatment is explained by random sampling of clonal isolates with higher or lower resistance and infectivity ranges at different time points. Two lines of evidence suggest that non-directional changes in average infectivity and resistance in this experimental system reflect range FSD rather than error or drift. First, a previous study employed DNA sequencing to demonstrate that changes in the frequencies of phage genotypes over time are consistent with positive selection for non-synonymous mutations (Hall *et al.* 2011). Second, if variation in average infectivity and resistance ranges over time reflected error in our sampling of clonal isolates from different time points, and not changes in their frequency in the population according to selection, individual phenotypes would appear and disappear in our samples across time randomly, rather than increasing and decreasing in frequency gradually, which was more typical in our data set (e.g. Fig. 5). Finally, drift and stochastic effects on the likelihood of cycles (Black & McKane 2012) could only explain the different coevolutionary dynamics between nutrient supply treatments if these processes had a greater effect in low nutrient treatments. This is highly unlikely, given the massive population sizes (in excess of 10^8^ bacteria) under both treatments, as well as in our model (∼ 10^5^). However, in the many host–parasite systems where population sizes are smaller than here, stochastic effects may well become relatively more important under low versus high nutrient conditions. This in turn is likely to further increase the difference in the extent of ARD between high and low nutrient conditions, as non-ARD dynamics will be driven by both selection (i.e. range FSD and specialism FSD) and increased stochasticity under low nutrient conditions.

In this study, coevolving communities were only exposed to a single type of nutrient environment (either high or low), yet environmental heterogeneity can play a crucial role in coevolutionary dynamics (Thompson 2005). Increased nutrient availability increases population size (Rosenzweig 1995), hence gene flow across environments that vary in nutrient supply is likely to result in populations from high nutrient environments (i.e. populations experiencing ARD) dominating the population as a whole (Hochberg & van Baalen 1998; Forde *et al.* 2004, 2007; Lopez-Pascua *et al.* 2010). By contrast, temporal variation in nutrient availability, particularly if fast, has recently been shown to impede ARD by constraining selective sweeps (Harrison *et al.* 2013). It is therefore possible that temporal variation in nutrient availability may shift coevolutionary dynamics towards FSD, while spatial variation shifts dynamics towards ARD.

In conclusion, change in a ubiquitous environmental variable, nutrient availability, predictability affect qualitative coevolutionary dynamics and hence the impact of coevolution on other evolutionary and ecological processes. For example, specialism (Hamilton *et al.* 1990) and potentially range FSD (Sasaki 2000) can favour the evolutionary maintenance of sexual reproduction, while ARD probably cannot (Parker 1994). These findings are likely to hold for a range of host–parasite systems in which increased infectivity and resistance ranges can evolve, as is the case for many plant–pathogen (Thompson & Burdon 1992) and bacteria–virus interactions (Flores *et al.* 2011). From an applied perspective, our results may have important implications for disease emergence. The evolution of parasites with broad genotypic host range are more likely to cause epidemics in new host populations, and potentially even adapt more readily to new species (Marston *et al.* 2012; Poullain & Nuismer 2012) but see Scanlan *et al*. (2013).
