# Supplementary figures and images for: Higher resources decrease fluctuating selection during host–parasite coevolution
Source: Ecol Lett. 2014 Aug 28;17(11):1380–8. doi: 10.1111/ele.12337 (PMC4257576; doi:10.1111/ele.12337)

**A. Arms race dynamics**

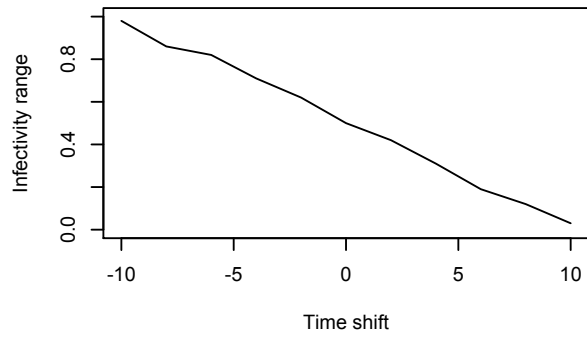

**B. Range fluctuating selection**

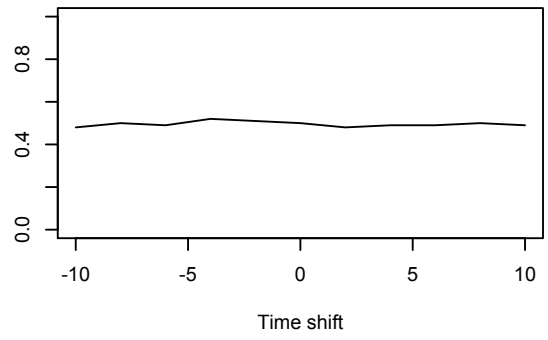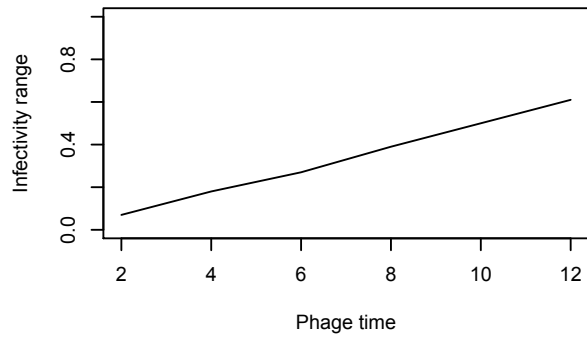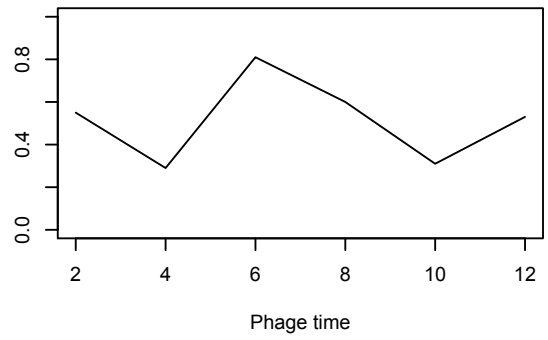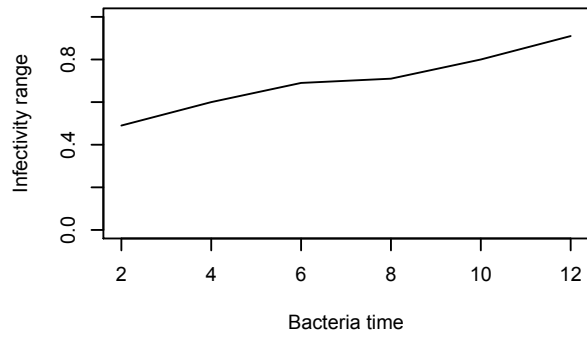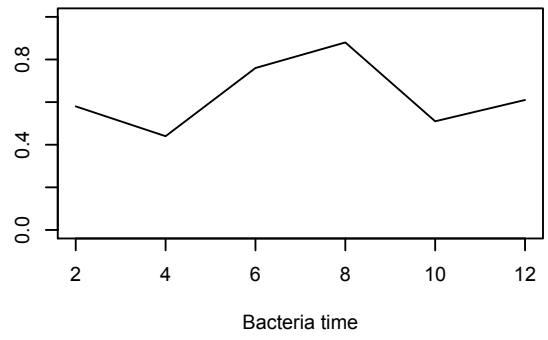

**C. Specialism fluctuating selection**

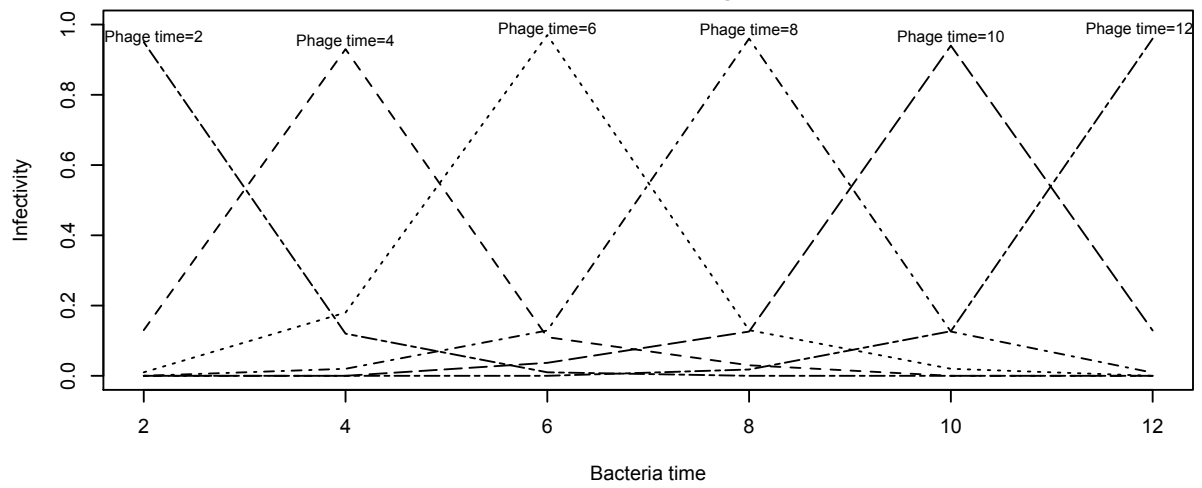

Supplement: Supplementary file 1 — Supplementary [file ele0017-1380-SD1.pdf]
