# Supplementary material for: Higher resources decrease fluctuating selection during host–parasite coevolution
Source: Ecol Lett. 2014 Aug 28;17(11):1380–8. doi: 10.1111/ele.12337 (PMC4257576; doi:10.1111/ele.12337)
Supplement: Supplementary file 3 — Supplementary [file ele0017-1380-SD3.docx]

**Supplementary material**

Table S1. Effects of phage time, bacteria time and time shift in every community.

| Nutrient supply | Community | MS_phage time_ | *P*_phage time_ | MS_bacteria time_ | *P*_bacteria time_ | MS_interaction_ | MS_time shift_ | *P*_time shift_ | Dynamics |
| --- | --- | --- | --- | --- | --- | --- | --- | --- | --- |
| 0.1 | a | 0.35 | <0.0001* | 0.08 | 0.0017* | 0.01 | 0.06 | 0.144 | rangeFSD |
| 0.1 | b | 0.04 | 0.1521 | 1.05 | <0.0001* | 0.02 | 4.73 | <0.0001* | ARD |
| 0.1 | c | 0.05 | 0.0126* | 0.16 | <0.0001* | 0.01 | 0.31 | 0.0029* | ARD |
| 0.1 | d | 0.13 | 0.0004* | 0.20 | <0.0001* | 0.02 | 0.01 | 0.65 | rangeFSD |
| 0.1 | e | 0.17 | <0.0001* | 0.47 | <0.0001* | 0.02 | 0.26 | 0.1 | rangeFSD |
| 0.1 | f | 0.09 | 0.0335 | 0.08 | 0.0569 | 0.03 | 0.04 | 0.297 | unknown |
| 1 | a | 0.05 | 0.392 | 0.20 | 0.0077* | 0.05 | 0.66 | 0.0015* | ARD |
| 1 | b | 0.02 | 0.033 | 0.14 | <0.0001* | 0.01 | 0.01 | 0.65 | rangeFSD_b_ |
| 1 | c | 0.02 | 0.55 | 0.33 | <0.0001* | 0.03 | 0.87 | 0.0004* | ARD |
| 1 | d | 0.29 | <0.0001* | 0.14 | 0.005* | 0.03 | 0.60 | <0.0001* | ARD |
| 1 | e | 0.02 | 0.0032* | 0.06 | <0.0001* | 0.00 | 0.24 | <0.0001* | ARD |
| 1 | f | 0.20 | <0.0001* | 0.30 | <0.0001* | 0.03 | 1.25 | <0.0001* | ARD |

Nutrient supply is given as relative nutrient concentration in liquid KB medium. Mean squares and *P*-values for phage time, bacteria time and the phage time×bacteria time interaction are from a model of infectivity in each population. Significance after sequential Bonferroni correction is indicated by an asterisk. The time shift effect was fitted in a model including phage time in each population; as noted by Rode et al., time shift and bacteria time cannot both be fitted alongside phage time because they are perfectly colinear within phage times. The coevolutionary dynamics indicated by the combination of main effects and time shift effect is given as ARD in cases where time shift was significant and FSD where main effects but not time shift were significant; range FSD_b_ indicates evidence of range FSD in bacteria only.

Figure S1. Schematic representation of coevolutionary dynamics. (**A**) Under arms race dyamics (ARD), average infectivity range of phages in a given population will decline with increasing time shift (bacteria time - phage time), and phages from later time points will have a higher average infectivity range than those from earlier time points. Similarly, under ARD bacteria from later time points will be resistant to a greater range of phages than bacteria from early time points. (**B**) By contrast, under range fluctuating selection (range FSD), there will be no directional change in average infectivity range with time shift, but both average infectivity and resistance will change significantly, but not directionally, across time points. (**C**) Under pure specialist fluctuating selection (specialist FSD), phages from each time point are specialised to infect bacteria from the same time point; note that this results in no average change in infectivity range of phages or resistance range of bacteria over time (no main effects), but does generate a significant phage time×bacteria time interaction.
